# Integrated efficacy analysis from phase 3 studies of investigational microbiome therapeutic, SER-109, in recurrent *Clostridioides difficile* infection

**DOI:** 10.1017/ash.2023.214

**Published:** 2023-09-29

**Authors:** Matthew Sims, Michael Silverman, Thomas Louie, Elaine Wang, Colleen Kraft, Mayur Ramesh, Tatiana Bogdanovich, Kelly Brady, David Lombardi, Asli Memisoglu, Ananya De, Brooke Hasson, Christine Lee, Paul Feuerstadt, Darrell Pardi, Colleen Kelly, Peter Daley, Godson Oguchi, Barbara McGovern, Lisa Von Moltke

## Abstract

**Background:** Antibiotics alone are often insufficient to treat recurrent *C. difficile* infection (rCDI) because they have no activity against *C. difficile* spores that germinate within a disrupted microbiome. SER-109, an investigational, oral, microbiome therapeutic comprised of purified *Firmicutes* spores, was designed to reduce rCDI through microbiome repair. We report an integrated efficacy analysis through week 24 for SER-109 from phase 3 studies, ECOSPOR III and ECOSPOR IV. **Methods:** ECOSPOR III was a randomized, placebo-controlled phase 3 trial conducted at 56 US or Canadian sites that included 182 participants with ≥2 CDI recurrences, confirmed via toxin EIA testing. Participants were stratified by age (<65 years or ≥65 years) and antibiotic regimen (vancomycin, fidaxomicin) and were randomized 1:1 to placebo or SER-109 groups. ECOSPOR IV was an open-label, single-arm study conducted at 72 US or Canadian sites including 263 participants with rCDI enrolled in 2 cohorts: (1) rollover participants from ECOSPOR III who experienced on-study recurrence diagnosed by toxin EIA (n = 29) and (2) participants with ≥1 CDI recurrence (diagnosed by PCR or toxin EIA), inclusive of the current episode (n = 234). In both studies, the investigational product was administered orally as 4 capsules over 3 consecutive days following symptom resolution after standard-of-care antibiotics. The primary efficacy end point was rCDI (recurrent toxin-positive diarrhea requiring treatment) through week 8. Other end points included CDI recurrence rates and safety through 24 weeks. **Results:** These 349 participants received at least 1 dose of SER-109 in ECOSPOR III or ECOSPOR IV (mean age 64.2; 68.8% female). Overall, 77 participants (22.1%) enrolled with their first CDI recurrence. Four participants received blinded SER-109 in ECOSPOR III followed by a second dose of open-label SER-109 in ECOSPOR IV. Overall, the proportion of participants who received any dose of SER-109 with rCDI at week 8 was 9.5% (33 of 349; 95% CI, 6.6 %–13.0%), and the CDI recurrence rate remained low through 24 weeks (15.2%, 53 of 349; 95% CI, 11.6%–19.4%), corresponding to sustained clinical response rates of 90.5% (95% CI, 87.0%–93.4%) and 84.8% (95% CI, 80.6%–88.4%), respectively (Fig. 1). Most rollover participants (25 of 29, 86.2%) were from the placebo arm; 13.8% had rCDI by week 8. **Conclusions:** In this integrated analysis, the rates of rCDI were low and durable in participants who received the investigational microbiome therapeutic SER-109, with sustained clinical response rates of 90.5% and 84.8% at weeks 8 and 24, respectively. These data further support the potential benefit of microbiome repair with SER-109 following antibiotics for rCDI to prevent recurrence in high-risk patients.

**Financial support:** This study was funded by Seres Therapeutics.

**Figure:**
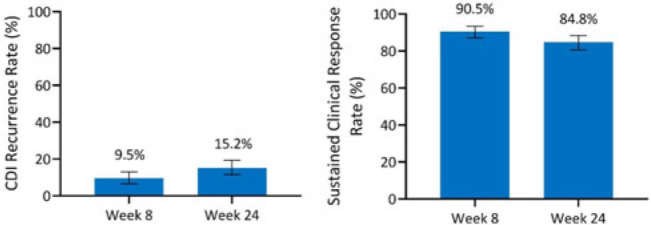

**Disclosure:** None

